# Comparing Eye Tracking with Electrooculography for Measuring Individual Sentence Comprehension Duration

**DOI:** 10.1371/journal.pone.0164627

**Published:** 2016-10-20

**Authors:** Jana Annina Müller, Dorothea Wendt, Birger Kollmeier, Thomas Brand

**Affiliations:** 1 Medizinische Physik and Cluster of Excellence Hearing4all, Universität Oldenburg, Oldenburg, Germany; 2 Hearing Systems, Department of Electrical Engineering, Technical University of Denmark, Lyngby, Denmark; 3 Eriksholm Research Centre, Snekkersten, Denmark; University of Pécs Medical School, HUNGARY

## Abstract

The aim of this study was to validate a procedure for performing the audio-visual paradigm introduced by Wendt et al. (2015) with reduced practical challenges. The original paradigm records eye fixations using an eye tracker and calculates the duration of sentence comprehension based on a bootstrap procedure. In order to reduce practical challenges, we first reduced the measurement time by evaluating a smaller measurement set with fewer trials. The results of 16 listeners showed effects comparable to those obtained when testing the original full measurement set on a different collective of listeners. Secondly, we introduced electrooculography as an alternative technique for recording eye movements. The correlation between the results of the two recording techniques (eye tracker and electrooculography) was r = 0.97, indicating that both methods are suitable for estimating the processing duration of individual participants. Similar changes in processing duration arising from sentence complexity were found using the eye tracker and the electrooculography procedure. Thirdly, the time course of eye fixations was estimated with an alternative procedure, growth curve analysis, which is more commonly used in recent studies analyzing eye tracking data. The results of the growth curve analysis were compared with the results of the bootstrap procedure. Both analysis methods show similar processing durations.

## Introduction

The human ability to comprehend speech is a complex process that involves the entire auditory system, from sensory periphery to central cognitive processing. Audiology uses different methods to assess the individual participant’s ability in speech comprehension. Pure-tone audiometry, for instance, primarily assesses sensory aspects, whereas speech audiometry assesses sensory as well as cognitive processes [1]. Taken by itself, speech audiometry does not enable a clear differentiation between sensory and cognitive mechanisms. However, speech audiometry may contribute to this differentiation when combined with additional measures that describe factors such as cognitive functions, speech processing effort, and processing duration [2, 3, 4]. Wendt et al. [5, 6] developed an audio-visual paradigm that uses eye fixations to determine the time required for sentence comprehension. They found a systematic dependence of the processing duration on sentence complexity, background noise, hearing impairment, and hearing aid experience. The ability to characterize the relative influence of peripheral auditory factors (by using conditions with and without background noise) that cause a reduction in speech comprehension and cognitive/central factors (by varying linguistic complexity) in listeners with impaired hearing makes this procedure potentially interesting for research and for clinical applications. However, the practical challenges required by Wendt et al. [5] were high: they employed an optical eye tracker and a measurement protocol consisting of up to 600 sentences per subject (requiring up to approximately three hours measurement time). This clearly limits the utility of this method.

The goal of this study was to evaluate comparatively more feasible alternatives to the method used by Wendt et al. [6], with regard to both the recording technique and the data analysis. Alternative methods were employed to investigate whether similar or even better information about processing duration in speech comprehension can be gained with fewer practical challenges. For that purpose, we evaluated a reduced set of sentences (around 400 instead of 600) from the Oldenburg Linguistically and Audiologically Controlled Sentences (OLACS; [7]) corpus. In addition, we compared two techniques for measuring eye fixation: eye tracking (ET) and electrooculography (EOG). Finally, we compared two analyzing strategies: the analysis method proposed by Wendt et al., 2015, which is based on a bootstrap procedure [8]; and the growth curve analysis (GCA) method developed by Mirman [9]. The former is considered standard for the audio-visual paradigm while the latter is more often used in recent studies analyzing eye tracking or pupillometry data [10, 11, 12].

The link between eye movements and speech processing was first discovered by Cooper [13]. Since then, a lot of research has investigated cognitive and perceptual processing based on eye movements and fixations (reviewed by [14]). For instance, Rayner [15] showed that eye fixation durations are affected by cognitive processes and that eye movement data possibly provide important and interesting information about human information processing. In a psycho-linguistic study, Tanenhaus et al. [16] used a visual world paradigm [17] to analyze acoustic speech processing and showed that visual context influenced spoken word recognition even during the earliest moments of language processing.

This indicates that an individual assessment of linguistic processing duration may be a valid measure for audiological assessment in addition to more peripheral measures of auditory performance, such as the pure-tone audiogram or speech comprehension in noise using only linguistically simple sentences. The audio-visual paradigm of Wendt et al. [5] applies a combination of acoustic and visual stimuli presented simultaneously. The acoustic stimuli of the OLACS corpus consist of different sentence structures that differ in their linguistic complexity, for instance, by using the canonical subject-verb-object (SVO) word order as opposed to the non-canonical and more complex object-verb-subject (OVS) word order. As visual stimuli, picture sets consist of two different pictures are shown on a computer screen. One picture shows the situation that is described acoustically, and the other illustrates the same characters with their roles reversed, so the agent (subject) is now the patient (object). An eye tracker records eye fixations during the participant’s task of selecting the picture that corresponds to the presented sentence as soon as possible by pressing a button. Based on the recorded eye fixations, the single target detection amplitude (sTDA) is determined; this describes the normalized rate of the participant’s fixations towards the target picture as a function of time. Based on the sTDA, the disambiguation to decision delay (DDD) is calculated as the period between the point in time at which the acoustic information that indicates the correct picture first becomes available and the actual time point of the participant’s decision, as indicated by his/her eye fixations. The DDD constitutes a measure of sentence processing duration.

Wendt et al. [5, 6] reported that processing duration is affected by several factors, including sentence complexity and background noise. Moreover, individual factors, such as cognitive abilities, hearing impairment, and hearing aid experience influence the time needed for processing a sentence [6, 18]. Therefore, they proposed the eye tracking paradigm as a potential tool for audiology and clinical application to monitor individual sentence processing duration. To facilitate this application, the current study proposes a method that is more feasible with respect to availability and cost of the measurement setup, robustness of the data evaluation procedure, and measurement time spent per patient. The aim of this study was to explore and evaluate alternative methods to those employed by Wendt et al. [6] on an individual basis testing listeners that differ in their hearing status.

The first alternative was the evaluation of a smaller measurement set, realized by reducing the number of sentences, in order to reduce the testing time. Fewer listening conditions (two instead of three) and fewer sentence structures (three instead of seven) were tested in the current study than by Wendt et al. [5].

As an alternative technique to eye tracking, electrooculography (EOG) was evaluated in this study within the paradigm proposed by Wendt et al. [5]. EOG records eye movements by measuring electrical potential differences between two electrodes. This takes advantage of the fact that the human eye is an electrical dipole consisting of a positively charged cornea and a negatively charged retina, first discovered by Schott in 1922 [19]. The potential difference between the poles is denoted as corneoretinal potential and lies in the range of 0.4 to 1 mV. Eye movements change the orientation of this dipole and the electric field, which can be recorded using two electrodes placed right and left besides the eyes for horizontal derivation and above and below the eyes for vertical derivation. The registered voltage changes proportionally to the angle of vision up to an angle of 30 degrees with an accuracy of 1.5 to 2 degrees [20]. In addition to diagnostic applications, EOG techniques are also applied in areas of human-machine interaction (e.g. [21, 22]).

Both EOG and ET are well established recording techniques for eye movements. Some studies already investigated the comparison of both recording techniques focusing on parameters such as saccade amplitude and velocity. Iacono et al. [23] investigated the quantitative and qualitative comparability of EOG recordings and infrared (IR) measures. They reported of high correlation coefficients ranging between .89 to .99 and thus indicating a high comparability between both recording techniques. Moreover, they proved high retest stability for the EOG within a single session, over a week, and over a two-year interval. In addition, Eggert et al. [24] did not find a significant difference in horizontal saccade amplitudes between IR and EOG recordings using a binocular setup for the EOG. Regarding the velocity of eye movements, Hess et al. [25] showed significant smaller peak velocities of saccades for the EOG, whereas Schmid-Priscoveanu et al. [26] did not find any differences in slow-phase velocity between both recording techniques. The advantages and disadvantages of EOG and ET devices are described and discussed extensively by Heide et al. [27]. One motivation for the evaluation of EOG in this study was to offer an alternative technique for registering eye fixations and eye movements in order to make the method cost-effective and feasible when no eye tracking device is available. Audiological centers often have access to EOG devices since EOG can very easily be integrated into multi-channel EEG setups.

The data analysis proposed by Wendt et al. [5] focuses on estimating processing duration by applying a bootstrap (BS) procedure to the normalized eye fixations in order to calculate single target detection amplitudes (sTDAs) and disambiguation to decision delays (DDDs). Alternatively, recent studies used growth curve analysis (GCA; [9]) as a tool for expressing the time course of eye fixations. GCA is a multilevel regression technique that estimates the time course of the data by fitting linear or higher-order polynomials. In order to investigate whether GCA would give more precise information or even additional information about individual processing duration, we calculated DDDs for both the BS and GCA methods, based on the estimated sTDAs, and compared these results. Moreover, we statistically compared the eye fixation time course between sentence structures and between listening conditions. Motivated by the fact that several studies successfully analyzed entire time courses using GCA (and not only a single point in time, as would be sufficient in our application), we compared results based on GCA with the results of the data analysis provided by Wendt et al. [6].

The following hypotheses were tested in this study:

1. The reduced set of sentences is sufficient to assess individual differences in sentence processing duration across listeners.
2. Both EOG and ET are suitable recording techniques for determining sentence processing duration. The two techniques provide similar estimates of sentence processing duration.
3. The data analysis developed by Wendt et al. [6] and GCA provide similar results for sTDA and processing duration.

## Materials and Methods

### Participants

17 participants (8 males) with an average age of 69 years (ranging from 55 to 75 years) participated in the experiment. Each participant conducted a hearing test under the ascending procedure in accordance with ISO 8253–1 [28] at frequencies from 125 Hz to 8 kHz (0.125, 0.250, 0.500, 0.750, 1, 1.5, 2, 3, 4, 6, and 8 kHz). Air-conducted and bone-conducted pure-tone hearing thresholds were measured to exclude participants with conductive hearing loss. The maximal difference of 20 dB between air- and bone-conducted thresholds was only accepted for two test frequencies on one ear. Five participants had pure-tone hearing thresholds of 20 dB hearing level (HL) or better at the audiometric frequencies in the range between 125 and 4000 Hz. Twelve participants had mild to moderate sensorineural hearing loss [29]. Their mean pure-tone average across all tested frequencies ranged from 35 dB HL to 49 dB HL, with a mean of 42 dB HL (SD = 10 dB HL). All seventeen participants were native German listeners and reported normal or corrected vision. Participants provided written informed consent. In addition, they were paid for participating in the experiment and were told that they could terminate their participation at any time. All participants met study criteria; but one hearing-impaired participant was unable to follow the instructions and was excluded from the final evaluation. The experiments and the consent procedure were approved by the local ethics committee of the University of Oldenburg.

### Material

#### Speech material: Oldenburg Linguistically and Audiologically Controlled Sentences (OLACS)

The OLACS corpus contains seven different sentence structures that vary in their linguistic complexity [7]. For the current study, we used three sentence structures from the OLACS corpus: subject-verb-object (SVO), object-verb-subject (OVS), and ambiguous object-verb-subject (ambOVS) sentences (Table 1). These sentence structures contain a transitive verb (i.e. an action verb which requires a subject and an object). The subject-first (SVO) sentence structure is the most commonly used structure in German [30]. The object-first (OVS) structure, however, is uncommon and syntactically complex due to the word order. Both SVO and OVS sentence structures are unambiguous: the article of the first noun of the sentence allows a clear assignment of agent (the character that is performing the action) and patient (the character that is affected by the action) roles. The third sentence structure is an ambiguous OVS structure, in which a clear assignment of agent and patient roles is made possible by the article of the second phrase. The word that disambiguates the sentence structure is underlined in Table 1. The point in time at which the disambiguating word starts and agent and patient roles can be assigned is denoted as the point of target disambiguation (PTD).

**Table 1:** OLACS sentence structures (SVO, OVS, and ambOVS).

**SVO**	Der kleine Junge grüsst den lieben Vater.
	The_nom_ little_nom_ boy_mal_ greets the_acc_ nice_acc_ father_mal_.
**OVS**	Den lieben Vater grüsst der kleine Junge.
	The_acc_ nice_acc_ father_mal_ greets the_nom_ little_nom_ boy_mal_.
**ambOVS**	Die nasse Ente tadelt der treue Hund.
	The_amb_ wet_amb_ duck_fem_ reprimants the_nom_ loyal_nom_ dog_mal_.

Nom (nominative), acc (accusative), and amb (ambiguous case) indicate the relevant case markings. fem indicates feminine gender and mal indicates male gender. Underlined words describe the point of target disambiguation (PTD). Parts of this table were adapted from Wendt et al. [5].

#### Visual stimuli

**Fig 1:**
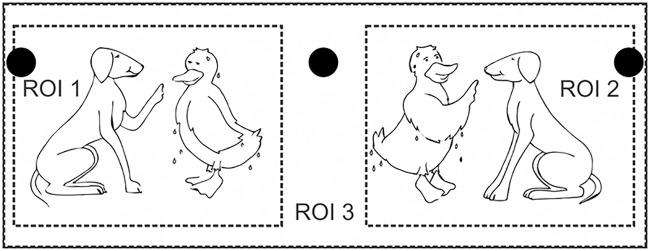
Visual stimulus. Picture set for the sentence: *Die nasse Ente tadelt der treue Hund*. In English: *The wet duck* (accusative, but indicated ambiguously by the article “die”, nominative would also be possible) *reprimands the loyal dog* (nominative, indicated by the unambiguous article “der”). The target is depicted on the left side and competitor on the right side in this example. The three black circles are the fixation points for the EOG calibration, which disappear when the picture set is displayed. ROI 1 and ROI 2 are the regions of interest where the pictures are displayed. ROI 3 is the background. The figure is adapted from Wendt et al. [5] in a modified version.

The audio-visual experiment used visual illustrations of the OLACS in the form of picture sets. All pictures and characters within the pictures were illustrated with the same size [5]. Each picture set consisted of two pictures presented side by side. The target picture illustrated the situation described by the spoken OLACS sentence, whereas the competitor picture illustrated the same characters in interchanged roles (Fig 1). For the analysis of the recorded eye fixations, the display screen was separated into three regions of interest (ROI): ROI 1 and ROI 2 were the regions for the two pictures and ROI 3 described the background of the screen (Fig 1). The target picture was randomly displayed in either ROI 1 or ROI 2.

### Methods

#### Speech reception threshold (SRT)

At the first session, the individual speech reception thresholds at 80% (SRT80) word recognition in modulated noise were assessed. Individual SRT80s were determined to perform the audio-visual paradigm at a defined high intelligibility. Modulated noise consisted of the standardized speech-shaped ICRA4 noise [31] with a female frequency spectrum and a maximal pause length of 250 ms (ICRA4-250 according to [32]). Sentences were presented via headphones. The participants were instructed to repeat the sentence as accurately as possible. Measurements in noise started at a SNR of 5 dB (speech signal 70 dB and noise signal 65 dB). The adaptive level adjustment (described as A1) developed by Brand and Kollmeier [33] was used to determine the level of the next sentence on the basis of the participant’s previous answer. All participants completed two training blocks in quiet and then one test block. The three sentence structures were presented randomly within each block that contained 60 sentences (20 SVO, 20 OVS, and 20 ambOVS). The SNRs of the last five trials were averaged to obtain the final SRT for each sentence structure individually.

#### Audio-visual paradigm

At the second and third session, the audio-visual paradigm was performed according to Wendt et al. [5]. The visual stimulus and the spoken sentence were presented together. The visual stimulus consisted of a picture set, as described above. Both pictures were presented starting 1000 ms before the acoustic sentence began. The participants’ task was to identify as quickly as possible the correct picture after sentence presentation by pressing one of three buttons on a gamepad: button one for the picture on the left side, button two for the picture on the right side, and button three for filler displays. About 9% of the sentences were filler displays; that is, neither the right nor the left picture matched the sentence. The filler displays forced the participants to consider both pictures, excluding the possibility of solving the task by looking only at one picture.

#### Recording eye fixations with eye tracking (ET) and electrooculography (EOG)

Eye fixations and eye movements were recorded using ET and EOG devices simultaneously. At the beginning of each test block, a nine-point fixation calibration was completed. Before the acoustic presentation of each single trial, a drift correction was realized: the participant was instructed to fixate a fixation point in the middle of the screen. For the EOG calibration, three additional fixation points were displayed successively on the left, on the right, and in the middle (Fig 1). The participants were asked to fixate these points for at least 500 ms. The EOG calibration was necessary in order to determine the ROIs with the EOG device. This procedure was completed before each trial to compensate for drift in the EOG signal. A chin rest was used in order to stabilize the participant’s head and keep it in the same position during the entire measurement.

Each participant performed 14 test blocks with altogether 405 sentences (135 of each sentence structure) in a randomized order in two sessions on two different days, which took about two hours. A training block consisting of 60 sentences was performed in quiet to allow the participant to become familiar with the task and with the material. Each sentence was presented in two listening conditions: in quiet at 65 dB (expected speech intelligibility of 100%) and in modulated noise at individual SRT80 (determined at the first session).

### Apparatus

#### Eye tracking

An eye tracker system (EyeLink 1000 desktop system including the EyeLink CL high-speed camera; SR Research Ltd., Mississauga, Canada) with a sampling rate of 1000 Hz was used to monitor participants’ eye movements. The pictures were presented on a 22″ multi-scan color computer screen with a resolution of 1680 x 1050 pixels. Participants were seated 60 cm from the computer screen.

#### Electrooculography

A pair of electrodes placed on the participant’s skin to the right and left of the eyes enabled the recording of horizontal eye movements. In addition, a ground electrode was placed on the participant’s nape. Before affixing the electrodes, the respective skin areas were cleaned and prepared with alcohol in order to reduce the skin resistance to below 10 kOhm. The potential difference between the electrodes was recorded using a BrainAmp DC EEG amplifier (Brain Products GmbH, Gilching, Germany) and the software Vision Recorder (Brain Products GmbH). All EOG signals were sampled at 5000 Hz. In order to synchronize EOG and ET, the soundcard generated the acoustically presented sentence using one stereo output channel, and a TTL signal using the other stereo output channel; this was conducted to one input channel of the EOG recorder. The acoustic stimulus was conducted to both sides of the headphones. Each time the EOG device received a TTL signal, a trigger was marked in the EOG raw data at the corresponding time. This was the case at the beginning and the end of the acoustic stimulus.

#### Setup for sound presentation

The two different measurements (SRT and audio-visual assessment of processing duration) took place in two different sound-isolated booths: During SRT measurements, experimenter and participant were seated in one booth together; during the audio-visual experiment, the participant was seated alone in a different booth. Sound presentation and signal adjustment were performed in the same way for both measurements. Sound stimuli were played via PC forwarded to an RME Digi96/8 PAD sound card (Audio AG, Haimhausen, Germany) and presented via HDA200 headphones (Sennheiser Electronic GmbH & Co. KG, Wedemark, Germany). The signals were adjusted using the NAL-R procedure [34] in order to consider participants’ individual hearing loss for listeners with hearing impairment. The signals were filtered with an 8-channel Butterworth filter bank (center frequencies: 0.25, 0.5, 0.75, 1, 2, 3, 4, and 6 kHz) in order to apply the required gain based on the individual audiogram. Afterwards, the signals were free-field equalized according to DIN EN ISO 389–8 (2004). Speech signals were calibrated with an artificial ear (4153), a ½ inch microphone (4134), a preamplifier (2669), and a measuring amplifier (2610) of Brüel & Kjaer (B&K, Naerum, Denmark).

### Data analysis

#### Eye fixations recorded with the eye tracking device

Fixations and saccades were obtained by the integrated software tool (Data Viewer) of the eye tracker. First, a heuristic filtering [35] removed one-sample spikes from the signal. Second, the EyeLink eye tracker used a saccade picker approach. This means that each data point that was not detected as a saccade or blink was defined as a fixation. Saccades were detected by computing the velocity and acceleration of each data point and comparing these values with fixed thresholds. A 17-sample velocity model calculated the velocity of a current data sample. Each sample with a velocity larger than 30°/sec or acceleration larger than 4000°/sec^2^ was detected as saccade.

#### Eye fixations recorded with the electrooculography device

Fixation and saccade information were obtained from the voltage signal recorded with the EOG device, using the Matlab Toolbox EEGLAB [36]. The above-mentioned EOG calibration and the saccade detection approach of the eye tracker were implemented in a Matlab script. The EOG device recorded one raw data set for each test block that was measured. Since ET was recorded with a sampling rate of 1000 Hz, we down-sampled the EOG data to match the ET sampling rate. Undesirable noises such as muscle activity, electrical interference, and high-frequency EEG influences the EOG signal, which was not the case for the ET signal. Therefore, these noise artifacts were removed from the EOG signal using a low-pass filter with a cutoff filter of 20 Hz. As the frequency spectrum of eye movements is limited to frequencies below 20 Hz, the recorded eye movements were not affected by the filtering. After this preprocessing, each test block of the EOG signal was divided into separate sentences on the basis of the recorded trigger. The following steps were executed for each sentence individually: Based on the EOG calibration, the horizontal boundaries of ROI 1 and ROI 2 were determined for each sentence individually in order to consider drift in the EOG signal. For this purpose, the EOG signal was averaged for 450 ms during the respective fixations towards the calibration points. In order to ensure a stable fixation towards the calibration points and to exclude overshoot after eye movement, only the last 450 ms of the 500 ms fixation period were considered. Subsequently each sample point was assigned to the ROIs: when the EOG signal was in the range between the averaging period of the left fixation point and the averaging period of the middle fixation point, the fixation was assigned to ROI 1; when the EOG signal was in the range between the averaging period of the right fixation point and the averaging period of the middle fixation point, the fixation was assigned to ROI 2. Points which did not fall in the range of ROI 1 or ROI 2 were assigned to ROI 3. In order to exclude saccades from the recorded signal, the same saccade picker approach of the eye tracker was realized for the EOG data analysis. The heuristic filtering [35] and a velocity and acceleration calculation based on 17-sample model (SR Research) were used with the same thresholds for velocity and acceleration.

#### Estimation of sentence processing duration based on a bootstrap (BS) procedure using eye fixation data

The estimation of individual sentence processing duration based on eye fixations is described briefly in the following. A more detailed description can be found in [6].

The processing was subdivided into three steps: the sentence-based processing stage, the sentence-structure-based processing stage, and the post-processing stage. In the first step, the fixations towards the three ROIs were analyzed for each sentence as a function of time. Since sentences differed in length, a time alignment of the recorded eye fixations was employed including a temporal segmentation of the eye movement data. Each trial (i.e. each presentation of a sentence and a picture set together with the participant’s response) was divided into 6 segments (Table 2). The segment borders were chosen according to the word that enables the identification of the target picture [5]. For the SVO and OVS sentence structures, the identification of the target picture is possible within segment 3, i.e. when the first noun is presented. For the ambOVS sentence structure, the identification is only possible within segment 4, i.e. with the presentation of the second article. In the second step, the time-aligned fixations were used to calculate fixation rates for the target and the competitor picture using a bootstrap (BS) procedure. BS performs a data resampling by randomly selecting 10000 fixations with replacement for each sample point. The single target detection amplitude (sTDA) was defined by subtracting the fixation rates of the competitor picture from the fixation rates of the target picture. The sTDA quantifies the tendency of the participant to fixate the target picture as a function of time: A positive sTDA means that the participant fixated the target picture more frequently; a negative sTDA means that the participant fixated the competitor picture more frequently.

**Table 2:** Segmentation of the stimulus presentation for statistical analysis.

	Segment 1	Segment 2	Segment 3	Segment 4	Segment 5	Segment 6
**Mean length /ms**	0–1000	1000–1745	1745–2340	2340–2995	2995–4140	4140–response
**SD**		130 ms	135 ms	130 ms	151 ms	114 ms
**Sentence**	Pictures	“*The wet duck reprimands the loyal dog*.”				Response

The pictures were presented alone during segment 1. During segments 2 to 5, the acoustical sentence was presented in addition to the pictures. In Segment 6 the pictures were still shown but the acoustical stimulus had ended and the participant was encouraged to respond. The mean length (in ms) and corresponding standard deviation (SD) of each segment were calculated over all sentences. The table is adapted from Wendt et al. [6] in a modified version.

In the third step, the 95% confidence interval was calculated based on the BS approach; this provides information about the variance of the sTDA. Only correctly analyzed trials were considered for the sTDA calculation. Based on the sTDA, the decision moment (DM) was calculated. The DM was defined as the point in time when the sTDA first reached a defined threshold for at least 200 ms. In order to calculate the processing duration, the disambiguation to decision delay (DDD) between the DM and the point of target disambiguation (PTD) was considered (Table 1). The averaged PTD was 1745 ms after the beginning of the sentence for SVO and OVS sentences and 2845 ms for ambOVS sentences.

#### Estimation of sentence processing duration based on the growth curve analysis (GCA) using eye fixation data

Growth curve analysis (GCA), as proposed by Mirman [9], is a multilevel regression technique that fits orthogonal polynomials to time course data in order to analyze differences between conditions and between individual participants. The GCA model consists of two hierarchically related submodels, where the level-1 submodel covers the effect of time:

$$Y_{ij}\;=\;\sum_{k\;=\;0}^{N}\beta_{ki}t_{j}^{k}+\epsilon_{ij},$$

where *Y*_*ij*_ is the individual response of participant *i* at time instant *j*, *N* the polynomial order, *ϐ*_*ki*_ the coefficient of time term *k* (intercept, linear, quadratic, etc.), and *ϵ*_*ij*_ the residual error.

Submodel level-2 considers individual random variations at different time terms (*ϐ*_*ki*_*)* and captures the effects of conditions. In this study this is the listening condition (quiet, noise) and the sentence structure (SVO, OVS, ambOVS):

$$\beta_{ki}\;=\;\gamma_{k0}+\;\gamma_{kC}C+\gamma_{kS}S+\zeta_{ki},$$

where *S* is the effect of sentence structure, *C* the effect of listening condition, *γ*_*k0*_ the baseline value, and *ζ*_*ki*_ the individual *i* random deviation.

To find the GCA model which, on the one hand, optimally estimates sTDAs and, on the other hand, investigates experimental effects on interpretable time terms, we followed the combined approach suggested by Mirman. This approach determines an upper limit to the polynomial order by the highest order that still improves the statistical fit, and a theoretical limit to the polynomial order by including only terms to the level-2 submodel, that have a meaningful interpretation. This resulted in a 10th order polynomial for the sTDA time course (level-1 model), since statistical comparison up to 10th order revealed an improvement in model fit compared to lower orders. To have interpretable results, we assumed the influence of listening conditions and sentence structure to be restricted to the first 5 polynomial orders (level-2 model). Random variations (*ζ*_*ki*_) were restricted to *ki* = 0 for *k* > 7 for numerical reasons, since models with higher order random effect structure did not converge.

Statistical significance (p-values) for individual parameter estimates was assessed using normal approximation. Analyses were performed using the software R Studio (RStudio, Boston, USA; Version 0.98.1103) with the package lme4.

#### Estimation of the DM

The data analysis developed by Wendt et al. [5] was modified with regard to the estimation of the DM: In order to take into account participants’ strategies, we introduced a relative threshold for the calculation of the DM which depends on the maximum of sTDA amplitude. The original threshold was defined at a fixed sTDA level of 15%. This value was chosen to account for small fluctuations in the sTDA that might not be relevant for speech processing. Moreover, it was shown that at this threshold the sTDA significantly differs from zero indicating more fixations towards the target picture [5]. Differences in absolute sTDA that occurred between participants were not taken into account using this fixed threshold value. In contrast, in this study the relative threshold was defined as 42% of the maximum sTDA averaged across sentence structures for each participant. This value was chosen because it best approximated the original definition of 15% (fixed level) when averaging across all participants.

#### Statistical analysis

In order to analyze the three initial hypotheses, the following statistical analyses were performed using the statistical software package IBM SPSS Statistics 21 (IBM Corporation, Armonk, USA). In order to investigate whether a reduced set of sentences was sufficient to reveal differences between sentence structures, we first tested the DDDs of all sentence structures for normal distribution with the Kolmogorov-Smirnov test. Since not all data showed a normal distribution, the non-parametric Friedman test was used. When the Friedman test revealed significant initial analysis, post hoc analysis was performed using the Wilcoxon test. Because there were multiple comparisons (DDD_SVO_ against DDD_OVS_; DDD_SVO_ against DDD_ambOVS_; DDD_OVS_ against DDD_ambOVS_), the significance level was adjusted, using the Bonferroni correction, to α = 0.0167. Cross correlations between sTDAs from eye fixations recorded with EOG and with ET and between BS and GCA were analyzed in order to investigate differences in sTDAs between eye movement recording techniques and analysis methods. In order to investigate statistical differences in DDDs between recoding techniques (ET and EOG) and analysis methods (BS and GCA), the Friedman test and subsequently the Wilcoxon test were used. Comprising statistical results of DDD differences are provided in the supporting information (S2 Table).

## Results

### Eye tracking and electrooculography

#### Effect of recording technique on single target detection amplitudes (sTDAs)

**Fig 2:**
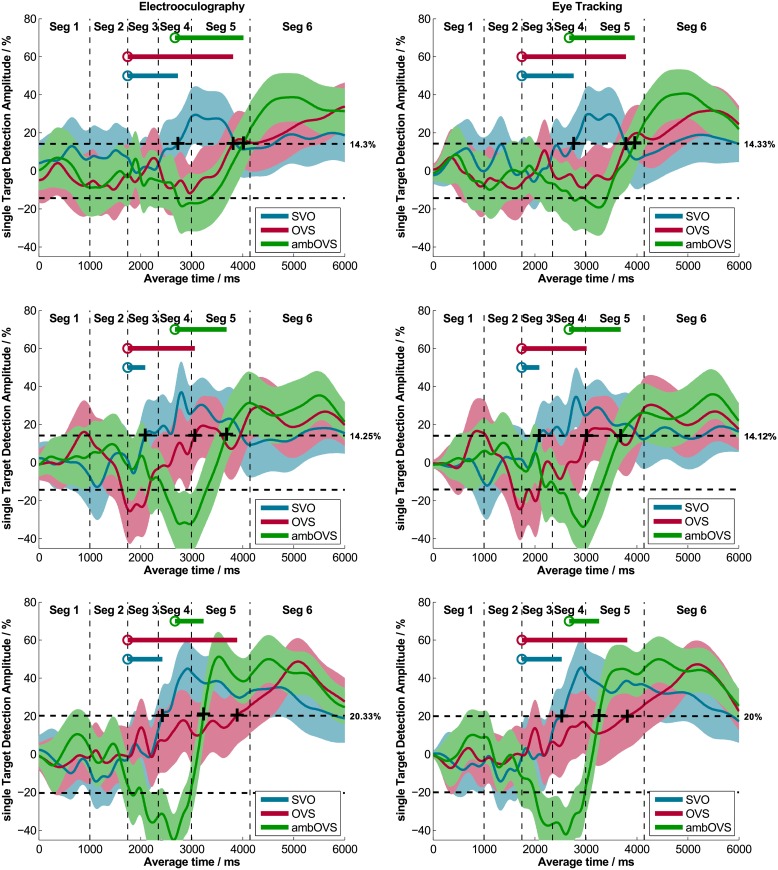
Single target detection amplitudes (sTDAs) recorded with EOG and ET. Three exemplary participants recorded simultaneously with EOG (left panel) and ET (right panel) are shown. The colored areas represent the 95% confidence intervals. The plus signs denote the DM where the sTDA first exceeds a relative threshold (indicated right beside the dashed line) and the circles describe the PTD for each sentence structure. The line starting from the PTD indicates the DDD, which represents the sentence comprehension processing duration.

sTDAs for each sentence structure of OLACS were calculated from EOG and ET data using bootstrapping (BS) for each participant in each listening condition. Fig 2 shows individual sTDAs for EOG and ET of three participants exemplarily. The shaded areas around the sTDAs illustrate the 95% confidence intervals calculated using BS. The panels on the left side show EOG data and the panels on the right side show ET data. The data of the two methods show very similar time courses; this was observed for all participants. Cross correlations between the sTDAs of the two recording techniques were calculated for each participant and each sentence structure in both listening conditions (quiet and noise). Averaged across all participants, sentence structures, and listening conditions, the calculated cross correlation was r = 0.97 (Table 3, left two panels). The highest individual cross correlation was r = 0.996 and the lowest individual cross correlation was r = 0.86 (individual data are provided in the supporting information; S3 Table). The two techniques produced nearly identical results in the time course of sentence processing.

**Table 3:** Cross correlations between sTDAs of the two recording techniques and analysis methods.

	Recording techniques (ET, EOG)		Analysis methods (BS, GCA)	
	quiet	mod. noise	quiet	mod. noise
**SVO**	0.975	0.973	0.959	0.957
**OVS**	0.972	0.966	0.959	0.946
**ambOVS**	0.981	0.979	0.967	0.963

Cross correlations r between sTDAs of both recording techniques (ET and EOG, left two panels) calculated using bootstrapping (BS) and cross correlations r between both analysis methods (BS and GCA, right two panels) based on EOG data for each listening condition (quiet, noise) and sentence structure (SVO, OVS, ambOVS) averaged across all participants.

#### Effect of recording technique on disambiguation to decision delays (DDDs)

**Fig 3:**
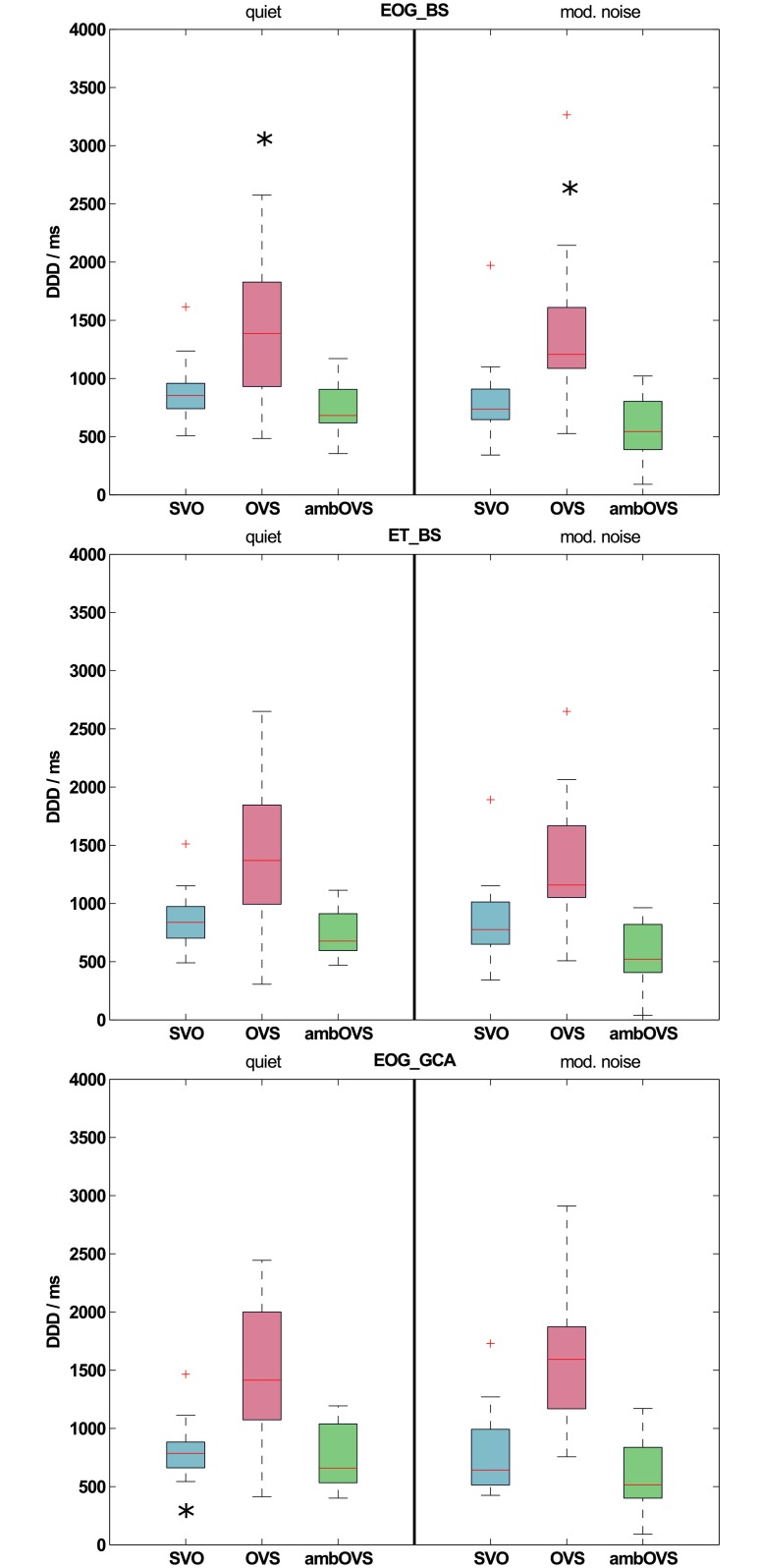
Disambiguation to decision delays (DDDs) across all participants. DDDs in quiet and modulated noise recorded with EOG and analyzed with the bootstrap procedure (EOG_BS; upper panel), recorded with ET and analyzed with the bootstrap procedure (ET_BS; middle panel), and recorded with EOG and analyzed with the growth curve analysis (EOG_GCA; lower panel) across all participants. Significant differences between sentence structures in the EOG_BS condition are denoted by an asterisk (*) above the sentence structure that differs from the other sentence structures (upper panel). Significant differences between sentence structures of different recording techniques (between EOG_BS and ET_BS) or analysis methods (between EOG_BS and EOG_GCA) are denoted by an asterisk (*) below the respective sentence structure in panel ET_BS and EOG_GCA.

**Fig 4:**
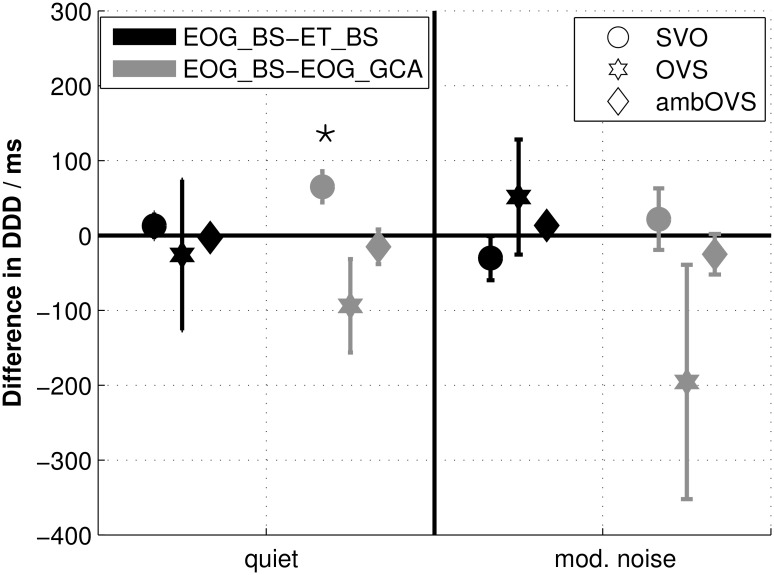
Disambiguation to decision delay (DDD) differences between recording techniques and analysis methods. Differences with standard errors between disambiguation to decision delays (DDDs) of data recorded with EOG and analyzed with the bootstrap procedure (EOG_BS) and data recorded with ET and analyzed with the bootstrap procedure (ET_BS) are indicated in black. Differences between DDDs of EOG_BS and data recorded with EOG and analyzed with the growth curve analysis (EOG_GCA) are indicated in grey. Statistical differences from zero are denoted by an asterisk (*) above the respective sentence structure.

Fig 3 shows boxplots of DDDs across all participants for both recording techniques determined with the BS procedure (EOG_BS: upper panel; ET_BS: middle panel). Statistical analysis revealed no differences between DDDs of both recording techniques for all sentence structures (all p > 0.05). Fig 4 shows the averaged differences in DDDs with standard errors between the two recording techniques (black symbols). Even though the OVS sentence structure showed larger standard errors and a slight difference from zero, DDD differences between recording techniques does not differ significantly from zero (all p > 0.05). These results clearly show that the DDDs of the two recording techniques are highly comparable.

#### Introduction of a relative decision moment (DM) threshold

**Fig 5:**
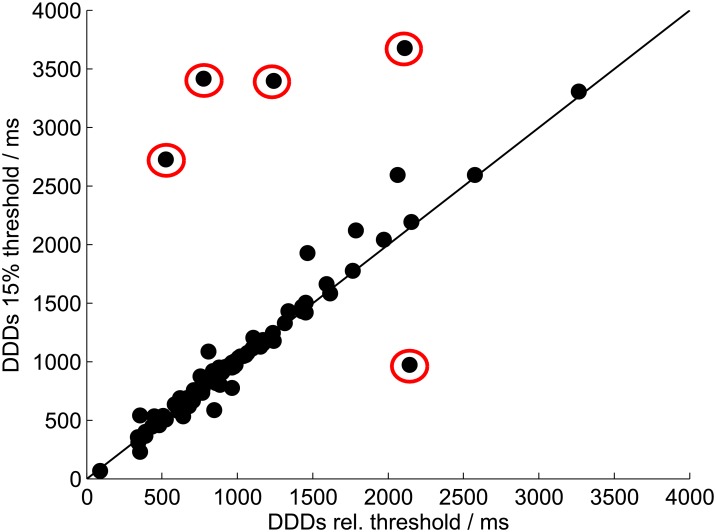
Relative threshold and fixed threshold. DDDs of the EOG_BS data determined based on the individual relative threshold compared to DDDs determined based on a fixed threshold of 15%. The results of all participants in both listening conditions are shown. Outliers are highlighted by red circles.

Fig 2 shows individual thresholds for each participant used to calculate the DM. These thresholds follow the definition of the DM introduced in this study, which considers differences in sTDA amplitudes. The range of thresholds across all participants, listening conditions, and recording techniques varies from 9.1% to 20.3%, with an average of 15.1%. The correlation between DDDs determined with the individual relative threshold and DDDs determined with a fixed threshold of 15% was r = 0.91 (p < 0.01; Fig 5). The five outliers highlighted in Fig 5 were from participants with either extremely low sTDAs (relative thresholds of 9.1%, 9.7%, and 11.7%) or extremely high sTDAs (relative threshold of 22.3%). Reasons for these large inter-individual differences in sTDAs are discussed below.

### Bootstrapping and growth curve analysis

#### Effect of analysis method on single target detection amplitudes (sTDAs)

**Fig 6:**
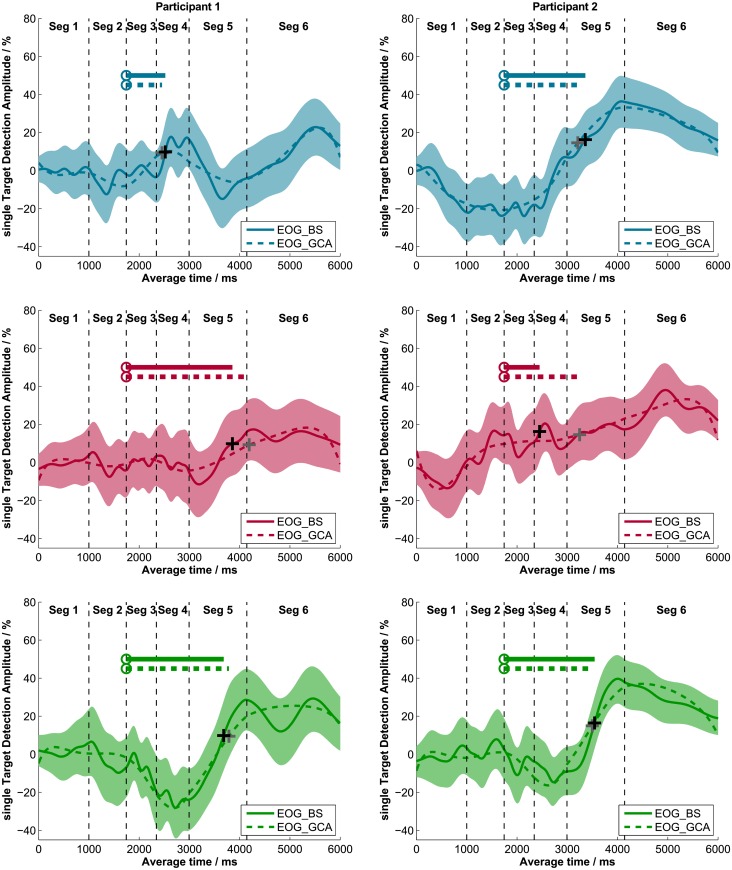
Single target detection amplitudes (sTDAs) of the BS and GCA. sTDAs determined with the bootstrap procedure (solid lines) and sTDAs modeled with the GCA procedure (dashed lines) of two exemplary participants (left and right panels). The three sentence structures are displayed separately (SVO: upper panels; OVS: middle panels; ambOVS: lower panels). The colored areas represent the 95% confidence interval of the bootstrap procedure. The plus signs (black: sTDA BS; grey: sTDA GCA) denote the DM where the sTDA first exceeded a relative threshold and the circles indicate the PTD for each sentence structure. The line starting from the PTD shows the DDD; this represents the processing duration during sentence comprehension (solid: DDD BS; dashed: DDD GCA).

Results of the original data analysis of the audio-visual paradigm developed by Wendt et al. [6], which uses a bootstrap (BS) procedure to calculate the sTDAs based on EOG data, were compared to results of the growth curve analysis (GCA). The GCA procedure modeled sTDAs with orthogonal polynomials based on fixation data. Fig 6 shows these polynomial fits as dashed lines and the sTDA calculated with the BS procedure as a solid line. The left panels and the right panels of Fig 6 show the respective data of one exemplary participant. Visual comparison of the time course of the two methods made it obvious that GCA fits the time course of the sTDA precisely. Cross correlations between sTDAs calculated with the BS and CGA method confirm the visual impression, that both methods produce similar sTDA time courses (Table 3, right two panels).

#### Effect of analysis method on disambiguation to decision delays (DDDs)

From the individual polynomial fits, the corresponding DDDs were calculated and compared to DDDs calculated from the sTDAs determined with the BS procedure. DDDs for both methods based on EOG data are shown in Fig 3 (EOG_GCA). DDDs calculated from the GCA were comparable to those calculated using BS. Only for the SVO sentence structure in quiet, DDDs of GCA differed significantly from DDDs of BS (quiet: z = -2.586, p = 0.01; all others p > 0.05). The differences between DDDs of EOG_BS and EOG_GCA are shown in Fig 4 (grey symbols). The DDD differences between analysis methods (EOG_BS and EOG_GCA) differed significantly from zero for SVO sentence structure in quiet (z = 2.586, p = 0.01).

#### GCA statistical model comparisons

Growth curve analysis according to [9] was conducted to analyze the time course of eye fixations measured with the audio-visual paradigm. Fixed effects of listening condition (quiet, noise) and sentence structure (SVO, OVS, and ambOVS) were considered in the level-2 submodel until the 5th order polynomial. The results show a significant effect between sentence structures on almost all time terms (p ≤ 0.05) except the slope. Significant differences on slope were only found between OVS and ambOVS in noise (Estimate = 42.98; SE = 14.82; p = 0.003) as well as an interaction effect between OVS and ambOVS in quiet relative to noise (Estimate = 42.46; SE = 20.95; p = 0.04). No differences were found between quiet and modulated noise for all sentence structures on all time terms (complete results of model comparisons are in S4 Table in the supporting information).

#### Effect of measurement set size

Averaged DDDs for each sentence structure determined from EOG recordings were compared statistically to investigate whether differences in processing durations between sentence structures, as revealed by Wendt et al. [6], are reproducible with a smaller measurement set and with a different collective of participants. Since no statistical differences were found between DDDs recorded with the ET and those recorded with the EOG, the analyses between sentence structures were conducted on the EOG data analyzed with the BS method. The greatest DDD was observed for the OVS sentence structure: 1386 ms in quiet and 1207 ms in noise, averaged across all participants. Smaller DDDs were found for SVO sentences (853 ms in quiet and 737 ms in noise) and ambOVS sentences (682 ms in quiet and 544 ms in noise). Both in quiet and in modulated noise, the DDDs of the OVS sentence structure differed significantly from the DDDs of SVO (quiet: z = -2.637, p = 0.006; noise: z = -3.154, p = 0.001) and ambOVS (quiet: z = -3.413, p < 0.001; noise: z = -3.413, p < 0.001) sentences (upper panel of Fig 3).

## Discussion

The audio-visual paradigm developed by Wendt et al. [5] enables the analysis of sentence comprehension processing duration. In this study, a reduced measurement set was tested, which reduced testing time. Furthermore, EOG was evaluated as an alternative eye fixation recording technique within the audio-visual paradigm in order to provide an alternative recording technique that can easily be integrated in an EEG setup. Furthermore, an alternative analysis method was investigated and tested for consistency.

### Reduction of the measurement set

In order to investigate the hypothesis that the reduced set of sentences is sufficient to assess individual differences in processing duration, we analyzed the data with regard to the effect of sentence structure. As reported by Wendt et al. [6], the DDD strongly depends on syntax complexity.

Processing duration was longer for the OVS structure than for the SVO structure (533 ms longer in quiet and 470 ms longer in noise). Longer durations probably arose from the additional cognitive processing cost of the more complex OVS structure. Previous studies also reported this increase in processing cost for more complex sentence structures (e.g. [30, 4]). The processing duration for the ambOVS structure was shorter than for the non-ambiguous OVS structure. Wendt et al. reported the same effect and argued that this was due to a change in the decision making process (see [5] for a more detailed discussion). In contrast to Wendt et al. [5], we only found significant differences between OVS and SVO sentences as well as OVS and ambOVS sentences, but no significant effect between SVO and ambOVS sentences. In quiet, this was due to the difference in DDDs for the ambOVS sentence structure between both studies. Wendt et al. [5] reported DDDs of about 500 ms for the ambOVS sentences, and we found a median DDD of around 682 ms. In modulated noise, the DDDs of the two studies were similar. This was also true for the DDDs of the OVS sentence structure (1400 ms averaged across HI and NH groups measured by Wendt et al. and 1386 ms measured in this study). It is assumed that the statistical differences observed between the two studies arose from using a different collective of participants, and not from the smaller measurement set. Had the smaller measurement set a significant impact, it would have influenced the processing duration for all sentence structures and not only some. The results of the present study indicate that a smaller measurement set can be used to detect sentence structure-dependent differences in processing duration, facilitating shorter measurement time.

### Comparison of recording techniques: eye tracking (ET) and electrooculography (EOG)

ET and EOG are standardized and reliable recording techniques for eye movements. We hypothesized that the two recording techniques would lead to very similar estimates of the sTDA curves and thus the processing duration. Cross correlations between sTDAs calculated from the data recorded with EOG and that recorded with ET were around 0.97, averaged across all participants; this indicated very similar sTDA curves for the two techniques. The small variations between the results may have arisen from different factors. Firstly, external disturbances affect the two recording techniques in different ways. For instance, changes in skin resistance influence the amplitude of EOG recordings, whereas ET recording remains unaffected [27]. Electrical noise influences the EOG signal but not the ET signal. Further, the EOG signal is not interrupted when eyes closed, whereas in ET, the reflection of infra-red light is interrupted by blinking, leading to a loss of signal. Small head movements are another factor. In order to prevent head movements, a chin rest was applied. However, small movements during measurement are not completely preventable. These movements affect the two recording techniques in different ways. Head-mounted recording devices, such as EOG, record the eye movements relative to the head. Desktop-mounted systems, such as the ET device used here, record the gaze relative to the table. These differences explain the slight variations in the sTDA curve shape.

In addition to comparing the sTDAs from the two recording techniques, DDDs were also calculated for each individual participant and for each sentence structure. In agreement with the very high cross correlations between sTDAs, no significant differences between the DDDs of the two recording techniques were observed. Overall, EOG and ET provided nearly identical processing duration results. The important advantages of the EOG method in contrast to the eye tracker are its cost efficiency and that the fact that EOG can easily be integrated in EEG setups.

### Fixed versus relative DM threshold

The definition of a relative DM threshold, which depends on the maximum of the sTDA, considers variations in sTDA amplitudes between participants. A relative threshold of 42% of the averaged sTDA maxima for the three sentence structures was applied. With this definition, the averaged threshold across all participants was 15.1%, which is very close to the original fixed definition of 15%. The large range of absolute sTDA thresholds, from 9.1% to 20.3%, illustrates the advantage of a relative definition. The comparison between relative and fixed thresholds showed large differences in DDDs for participants with extremely high or extremely low sTDAs. A fixed threshold, as used in [6], cannot consider these amplitude differences between participants. Amplitude differences arose from different strategies participants used to solve the task of the audio-visual paradigm. Some participants switched between the pictures and visually inspected both pictures for the same amount of time. Others preferred to visually inspect one picture in more detail and gave the other picture just a short look to identify filler displays and to confirm their decision. These differences in strategies led to different sTDA amplitudes, which influenced the calculation of processing duration. Even though we found a very high correlation, of r = 0.91, between DDDs of the individual relative threshold and a fixed threshold of 15%, some participants showed strong differences in DDDs (Fig 5; outliers highlighted in red). As a result, all of those outlier threshold values derived from the fixed threshold procedure, which were outside of the expected DDD range (marked as red dots above the diagonal line in Fig 5) were within the normal, expected range when the relative threshold criterion was used.

Wendt et al. [6] was forced to exclude some individual participant data because the sTDAs did not reach the threshold of 15%. With the new DM definition, the analysis of these data would be possible. Based on the results presented here and by Wendt et al. [6], we strongly recommend applying the relative threshold instead of a fixed threshold definition.

### Comparison of analysis methods: bootstrapping (BS) and growth curve analysis (GCA)

The data analysis of the audio-visual paradigm uses a bootstrap procedure (BS) for the calculation of the 95% confidence interval and the median value of the sTDA [6]. One goal of the current study was to examine whether GCA provides reliable estimates of the sTDA, and thus the processing duration, similar to those obtained using the BS procedure. The time course of sTDA was modeled with a 10th order polynomial. This high polynomial order was necessary to optimally track the time course of the sTDA, since sTDAs changed directions many times over the whole time course. Other studies, which analyzed their data with GCA used lower order polynomials, due to less complex data [37, 38, 39]. The 10th order polynomial model shaped the sTDA time courses very well and supported our hypothesis that the two methods provide similar results. High cross correlations between sTDAs of both methods confirmed this finding. Estimated DDDs were very similar to DDDs calculated with the original BS data analysis. Only DDDs of the SVO sentence structure in quiet differed significantly between both analysis methods. About 64 ms smaller processing durations were calculated with the GCA for the SVO sentence structure in quiet. This small difference did not influence the interpretation of the overall results. The processing duration for the OVS sentence structure was still the longest, followed by SVO and ambOVS sentences.

Additional desired properties of GCA are that it could provide information about eye fixation time course data with a low number of parameters and that it allows statistical model comparisons. This may help to characterize information about differences between listening conditions and sentence structures on different time terms (e.g. intercept, linear, quadratic). Here, we considered time terms until the 5th polynomial, since these provide interpretable and interesting results for sTDA data. The statistical analysis revealed an effect of sentence structure, i.e. there is a significant difference between the eye fixation time courses of all three sentence structures on the intercept, which reflects the average of the whole time course. SVO showed the highest averaged sTDA time course, followed by OVS, and ambOVS. This, at the first glance, indicates an earlier fixation of the correct picture and therefore the fastest processing duration for the SVO sentence structure, followed by OVS, and ambOVS. But this result is not in line with the analysis of DDDs between sentence structures, which revealed the fastest processing for the ambOVS sentence structures, followed by SVO and OVS. The mentioned differences are likely due to differences in the analysis strategies. The statistical GCA analysis considered the whole time course of sTDAs, whereas by determining differences between DDDs, we focused on one specific point in time. This difference of analysis strategies leads to different interpretations. If we consider information about the slope of the sTDA time courses, no differences can be found between sentence structures, except between OVS and ambOVS in noise. This demonstrates no differences in the angles between sTDAs, indicating similar fixations rates towards the target picture. Results of the higher polynomial orders are already very challenging to interpret. Kuchinsky et al. [10] reported that the quadratic term reflects the shape of the central inflection. The cubic term indicates an earlier or later peak of the response. Quartic and quantic terms give information about the secondary peaks of the time course. Considering these time terms on our data, we found statistical differences on almost all terms for all sentence structures in both listening conditions. This indicates significant differences in the eye fixation time course between sentence structures in both listening conditions. The GCA data analysis revealed strong differences in eye fixations regarding sentence structures. However, we could not find a difference between listening conditions at all. In other words, no significant differences were found when participants were listening to the sentence in quiet or in noise. GCA provides an easy and statistical appropriate way to analyze differences between the processing of different complex sentence structures. It does not only consider DDDs but also the whole time course of fixations. However, GCA is not as good as the BS procedure at estimating the statistical uncertainty at a single point in time: The purpose of the method by Wendt et al. [5] was to investigate the processing duration based on a specific point in time at which the participant made their decision; this time point was indicated by their eye fixations and was defined as the DM. The BS also provides, besides the estimated median, the 95% confidence interval of the sTDA, which is especially important at that point of interest, since it indicates how certain the calculated DM was. Since we were mostly interested in a specific point in time, and since both analysis methods provided very similar DDDs, the BS procedure is the recommended method because it also provides the confidence interval of the data. One way to improve the GCA data analysis for this specific application could be to only consider a specific part of the sTDA time course and not the whole time. However, GCA can be used as an alternative analysis method to detect the processing duration, or if there is more interest in the actual time course of the eye fixations during sentence recognition.

## Conclusion

This study evaluated possible improvements on the audio-visual paradigm to extract individual differences in linguistic processing duration of sentences with varying linguistic complexity, developed by Wendt et al. [5]. The following aspects appear to be important for these purposes:

1. Even with a reduced measurement set (fewer sentences and fewer listening conditions) differences in processing durations between sentence structures could be detected.
2. The EEG-setup-based electrooculogram (EOG) method for recording eye fixations was successfully evaluated as a reliable and suitable alternative to the eye tracking (ET) technique used in the paradigm proposed by Wendt et al. [5]. Using a nearly identical data analysis, the EOG technique applied in the audio-visual paradigm enables the investigation of processing duration during sentence comprehension.
3. A new definition of the individual relative threshold for the decision moment (DM) was introduced. This definition considers differences in participants’ strategies. This enables a more robust data analysis.
4. The data analysis developed by Wendt et al. [5] and the polynomial fit of the growth curve analysis (GCA) provided almost identical processing duration results for individual participants. Both analysis methods are recommended, depended on the users’ interest.

## Supporting Information

S1 Table Individual processing duration (DDD). (DOCX)

S2 Table Statistical results comparing DDDs. (DOCX)

S3 Table Cross-correlations between sTDAs of both recording techniques (ET and EOG) and of both analyzing methods (BS and GCA). (DOCX)

S4 Table Model’s parameter estimates. (DOCX)
