# Supplementary material for: Comparing Eye Tracking with Electrooculography for Measuring Individual Sentence Comprehension Duration
Source: PLoS One. 2016 Oct 20;11(10):e0164627. doi: 10.1371/journal.pone.0164627 (PMC5072642; doi:10.1371/journal.pone.0164627)
Supplement: S1 Table — (DOCX) [file pone.0164627.s001.docx]

S1A Table. Individual processing duration (DDD) recorded with EOG and analyzed with bootstrapping.

|  | **EOG_BS** | | | | | |
| --- | --- | --- | --- | --- | --- | --- |
|  | **quiet** | | | **mod. noise** | | |
|  | **SVO** | **OVS** | **ambOVS** | **SVO** | **OVS** | **ambOVS** |
| **S1** | 775.68 | 2109.65 | 837.24 | 853.49 | 1106.36 | 917.70 |
| **S2** | 1236.03 | 1431.51 | 975.17 | 853.49 | 1171.19 | 768.28 |
| **S3** | 769.20 | 1592.42 | 480.93 | 963.71 | 1041.52 | 388.98 |
| **S4** | 827.55 | 1339.56 | 618.86 | 448.54 | 1242.52 | 354.50 |
| **S5** | 847.00 | 885.91 | 618.86 | 678.42 | 2144.13 | 388.98 |
| **S6** | 898.87 | 2575.09 | 1055.63 | 585.12 | 1764.83 | 906.21 |
| **S7** | 671.94 | 859.97 | 354.50 | 704.36 | 1151.74 | 90.65 |
| **S8** | 1615.41 | 704.36 | 699.32 | 983.16 | 1171.19 | 630.35 |
| **S9** | 859.97 | 976.68 | 768.28 | 684.91 | 1454.50 | 480.93 |
| **S10** | 963.71 | 989.65 | 526.91 | 769.20 | 1067.45 | 434.96 |
| **S11** | 983.16 | 2063.68 | 1170.57 | 1099.87 | 3265.72 | 1021.15 |
| **S12** | 507.92 | 1465.99 | 1009.65 | 808.10 | 525.74 | 756.79 |
| **S13** | 950.74 | 484.17 | 664.83 | 652.49 | 1454.50 | 607.36 |
| **S14** | 607.10 | 1242.52 | 630.35 | 639.52 | 963.71 | 343.00 |
| **S15** | 879.42 | 2155.63 | 837.24 | 341.65 | 1316.57 | 837.24 |
| **S16** | 710.84 | 1431.51 | 641.85 | 1971.72 | 1787.82 | 457.94 |

Individual processing durations for all 16 participants recorded with EOG and analyzed with the bootstrap procedure for all three sentence structures in both listening conditions.

S1B Table. Individual processing duration (DDD) recorded with ET and analyzed with bootstrapping.

| **ET_BS** | | | | | |
| --- | --- | --- | --- | --- | --- |
| **quiet** | | | **mod. noise** | | |
| **SVO** | **OVS** | **ambOVS** | **SVO** | **OVS** | **ambOVS** |
| 730.29 | 2190.11 | 871.72 | 573.25 | 1106.36 | 929.19 |
| 1151.74 | 1431.51 | 952.18 | 821.07 | 1022.07 | 722.30 |
| 814.58 | 1626.91 | 503.92 | 944.26 | 1022.07 | 377.49 |
| 840.52 | 1362.55 | 664.83 | 519.80 | 1695.87 | 423.46 |
| 814.58 | 983.16 | 630.35 | 782.16 | 2063.68 | 411.97 |
| 1112.84 | 2649.75 | 1090.11 | 691.39 | 1787.82 | 825.75 |
| 646 | 853.49 | 469.44 | 769.20 | 1112.84 | 38.77 |
| 1511.97 | 1707.36 | 687.82 | 1145.26 | 1638.40 | 630.35 |
| 853.49 | 1002.61 | 722.30 | 717.33 | 1203.61 | 446.45 |
| 931.29 | 931.29 | 561.39 | 795.13 | 1048.00 | 423.46 |
| 1015.58 | 2040.69 | 1113.10 | 1151.74 | 2649.75 | 963.68 |
| 490.11 | 1983.22 | 975.17 | 1080.42 | 507.92 | 814.25 |
| 911.84 | 306.02 | 653.34 | 678.42 | 1557.94 | 595.87 |
| 567.31 | 1177.68 | 630.35 | 620.07 | 1067.45 | 331.51 |
| 834.03 | 1106.36 | 860.23 | 341.65 | 1270.59 | 837.24 |
| 671.94 | 1374.04 | 549.89 | 1891.27 | 1054.49 | 400.47 |

Individual processing durations for all 16 participants recorded with ET and analyzed with the bootstrap procedure for all three sentence structures in both listening conditions.

S1C Table. Individual processing duration (DDD) recorded with EOG and analyzed with growth curve analysis.

|  | **EOG_GCA** | | | | | |
| --- | --- | --- | --- | --- | --- | --- |
|  | **quiet** | | | **mod. noise** | | |
|  | **SVO** | **OVS** | **ambOVS** | **SVO** | **OVS** | **ambOVS** |
| **S1** | 710.84 | 2444.43 | 940.69 | 454.48 | 1534.96 | 710.81 |
| **S2** | 1112.84 | 1328.06 | 1044.13 | 834.03 | 756.23 | 814.25 |
| **S3** | 633.04 | 1569.44 | 526.91 | 1067.45 | 1132.29 | 423.46 |
| **S4** | 788.65 | 1500.47 | 538.40 | 525.74 | 1649.89 | 377.48 |
| **S5** | 691.39 | 931.29 | 664.83 | 620.07 | 2052.18 | 526.91 |
| **S6** | 918.32 | 2397 | 1090.11 | 613.58 | 2029.19 | 1021.15 |
| **S7** | 597 | 905.36 | 400.47 | 478.23 | 1203.61 | 90.65 |
| **S8** | 1465.99 | 1500.47 | 653.34 | 1112.84 | 1305.08 | 687.82 |
| **S9** | 782.16 | 1086.90 | 710.81 | 501.99 | 1649.89 | 503.92 |
| **S10** | 996.13 | 1060.97 | 480.93 | 898.87 | 1028.55 | 503.92 |
| **S11** | 840.52 | 2351.02 | 1136.08 | 1270.59 | 2911.07 | 1170.57 |
| **S12** | 555.43 | 1707.36 | 1193.55 | 918.32 | 2892.41 | 860.23 |
| **S13** | 847.00 | 412.91 | 630.35 | 573.25 | 1718.86 | 377.49 |
| **S14** | 543.55 | 1151.74 | 584.37 | 665.46 | 996.13 | 331.51 |
| **S15** | 743.26 | 2293.55 | 1032.64 | 424.79 | 1203.61 | 952.18 |
| **S16** | 840.52 | 1171.19 | 503.92 | 1730.35 | 1695.87 | 434.96 |

Individual processing durations for all 16 participants recorded with EOG and analyzed with growth curve analysis for all three sentence structures in both listening conditions.
