# Supplementary material for: Comparing Eye Tracking with Electrooculography for Measuring Individual Sentence Comprehension Duration
Source: PLoS One. 2016 Oct 20;11(10):e0164627. doi: 10.1371/journal.pone.0164627 (PMC5072642; doi:10.1371/journal.pone.0164627)
Supplement: S2 Table — (DOCX) [file pone.0164627.s002.docx]

S2 Table. Statistical results comparing DDDs.

|  | **EOG_BS vs. ET_BS** | | | | | | **EOG_BS vs. EOG_GCA** | | | | | |
| --- | --- | --- | --- | --- | --- | --- | --- | --- | --- | --- | --- | --- |
|  | **quiet** | | | **mod. noise** | | | **quiet** | | | **mod. noise** | | |
|  | **Z** | **p** | **r** | **Z** | **p** | **r** | **Z** | **p** | **r** | **Z** | **p** | **r** |
| **SVO** | -1.708 | .091 | -.43 | -1.506 | .139 | -.38 | -2.586 | .008 | -.65 | -.052 | .980 | -.01 |
| **OVS** | -0.511 | .639 | -.13 | -0.795 | .454 | -.20 | -1.345 | .188 | -.34 | -1.138 | .269 | -.28 |
| **ambOVS** | -0.313 | .772 | -.07 | -1.165 | .257 | -.29 | -.545 | .604 | -.14 | -1.535 | .135 | -.38 |

Z-score, p-value, and effect size r for statistical analysis between DDDs for all three sentence structures in both listening conditions. DDDs recorded with EOG and analyzed with bootstrapping are compared to DDDs recorded with ET and DDDs recorded with EOG and modeled with GCA.
