# Supplementary material for: Comparing Eye Tracking with Electrooculography for Measuring Individual Sentence Comprehension Duration
Source: PLoS One. 2016 Oct 20;11(10):e0164627. doi: 10.1371/journal.pone.0164627 (PMC5072642; doi:10.1371/journal.pone.0164627)
Supplement: S3 Table — (DOCX) [file pone.0164627.s003.docx]

S3 Table. Cross-correlations between sTDAs of both recording techniques (ET and EOG) and of both analysis methods (BS and GCA).

|  | **Recording techniques (ET, EOG)** | | | | | | **Analysis methods (BS, GCA)** | | | | | |
| --- | --- | --- | --- | --- | --- | --- | --- | --- | --- | --- | --- | --- |
|  | **quiet** | | | **mod. noise** | | | **quiet** | | | **mod. noise** | | |
|  | **SVO** | **OVS** | **ambOVS** | **SVO** | **OVS** | **ambOVS** | **SVO** | **OVS** | **ambOVS** | **SVO** | **OVS** | **ambOVS** |
| **S1** | 0.97 | 0.95 | 0.96 | 0.98 | 0.99 | 0.97 | 0.83 | 0.88 | 0.94 | 0.83 | 0.80 | 0.93 |
| **S2** | 0.99 | 0.99 | 0.98 | 0.97 | 0.99 | 0.99 | 0.98 | 0.95 | 0.97 | 0.98 | 0.97 | 0.97 |
| **S3** | 0.97 | 0.95 | 0.97 | 0.99 | 0.95 | 0.98 | 0.96 | 0.97 | 0.97 | 0.96 | 0.97 | 0.95 |
| **S4** | 0.99 | 0.99 | 0.98 | 0.98 | 0.96 | 0.99 | 0.95 | 0.92 | 0.98 | 0.87 | 0.91 | 0.95 |
| **S5** | 0.98 | 0.99 | 0.98 | 0.98 | 0.99 | 0.99 | 0.99 | 0.98 | 0.98 | 0.98 | 0.98 | 0.96 |
| **S6** | 0.96 | 0.96 | 0.99 | 0.96 | 0.96 | 0.96 | 0.98 | 0.93 | 0.95 | 0.99 | 0.95 | 0.95 |
| **S7** | 0.96 | 0.96 | 0.98 | 0.96 | 0.95 | 0.98 | 0.99 | 0.98 | 0.99 | 0.98 | 0.97 | 0.97 |
| **S8** | 0.98 | 0.97 | 0.98 | 0.99 | 0.95 | 0.98 | 0.99 | 0.97 | 0.95 | 0.98 | 0.98 | 0.97 |
| **S9** | 0.99 | 0.99 | 0.99 | 0.99 | 0.99 | 0.99 | 0.94 | 0.98 | 0.97 | 0.97 | 0.94 | 0.98 |
| **S10** | 0.98 | 0.97 | 0.99 | 0.99 | 0.97 | 0.99 | 0.96 | 0.99 | 0.98 | 0.98 | 0.96 | 0.96 |
| **S11** | 0.98 | 0.97 | 0.96 | 0.97 | 0.98 | 0.97 | 0.97 | 0.97 | 0.97 | 0.96 | 0.94 | 0.98 |
| **S12** | 0.92 | 0.95 | 0.99 | 0.95 | 0.97 | 0.97 | 0.97 | 0.97 | 0.94 | 0.95 | 0.95 | 0.95 |
| **S13** | 0.99 | 0.98 | 0.98 | 0.98 | 0.98 | 0.99 | 0.97 | 0.97 | 0.97 | 0.96 | 0.91 | 0.97 |
| **S14** | 0.99 | 0.99 | 0.99 | 0.99 | 0.99 | 0.99 | 0.98 | 0.99 | 0.98 | 0.98 | 0.99 | 0.98 |
| **S15** | 0.96 | 0.95 | 0.96 | 0.90 | 0.86 | 0.94 | 0.95 | 0.95 | 0.94 | 0.97 | 0.94 | 0.96 |
| **S16** | 0.99 | 0.99 | 0.99 | 0.99 | 0.99 | 0.99 | 0.95 | 0.96 | 0.98 | 0.97 | 0.98 | 0.95 |
