# Supplementary material for: Comparing Eye Tracking with Electrooculography for Measuring Individual Sentence Comprehension Duration
Source: PLoS One. 2016 Oct 20;11(10):e0164627. doi: 10.1371/journal.pone.0164627 (PMC5072642; doi:10.1371/journal.pone.0164627)
Supplement: S4 Table — (DOCX) [file pone.0164627.s004.docx]

S4A Table. Model’s parameter estimates for OVS in comparison to SVO.

|  | **OVS vs. SVO in quiet** | | | | **OVS vs. SVO in noise** | | | |
| --- | --- | --- | --- | --- | --- | --- | --- | --- |
|  | **Est.** | **SE** | **t** | **p** | **Est.** | **SE** | **t** | **p** |
| **Int.** | -1.92 | 0.94 | -2.05 | 0.041 | -2.58 | 0.94 | -2.75 | 0.006 |
| **ot1** | 0.23 | 14.82 | 0.016 | 0.99 | -20.26 | 14.82 | -1.37 | 0.17 |
| **ot2** | 64.57 | 23.78 | 2.72 | 0.006 | 90.88 | 23.78 | 3.82 | 0.0001 |
| **ot3** | 84.86 | 20.64 | 4.11 | 3.91e-05 | 70.67 | 20.64 | 3.43 | 0.0006 |
| **ot4** | -16.39 | 22.23 | -0.74 | 0.46 | -49.81 | 22.23 | -2.24 | 0.02 |
| **ot5** | -73.94 | 16.84 | -4.39 | 1.13e-05 | -34.94 | 16.84 | -2.08 | 0.038 |

S4B Table. Model’s parameter estimates for ambOVS in comparison to SVO.

|  | **ambOVS vs. SVO in quiet** | | | | **ambOVS vs. SVO in noise** | | | |
| --- | --- | --- | --- | --- | --- | --- | --- | --- |
|  | **Estimate** | **SE** | **t** | **p** | **Estimate** | **SE** | **t** | **p** |
| **Int.** | -7.12 | 0.94 | -7.58 | 3.51e-14 | -7.15 | 0.94 | -7.61 | 2.80e-14 |
| **ot1** | 0.76 | 14.82 | 0.05 | 0.96 | 22.72 | 14.82 | 1.53 | 0.13 |
| **ot2** | 259.02 | 23.78 | 10.89 | < 2.22e-16 | 243.54 | 23.78 | 10.24 | < 2.22e-16 |
| **ot3** | 151.79 | 20.64 | 7.36 | 1.90e-13 | 96.99 | 20.64 | 4.70 | 2.60e-06 |
| **ot4** | -133.23 | 22.23 | -5.99 | 2.06e-09 | -175.12 | 22.23 | -7.88 | 3.33e-15 |
| **ot5** | -177.82 | 16.84 | -10.56 | < 2.22e-16 | -141.12 | 16.84 | -8.38 | < 2.22e-16 |

S4C Table. Model’s parameter estimates for ambOVS in comparison to OVS.

|  | **ambOVS vs. OVS in quiet** | | | | **ambOVS vs. OVS in noise** | | | |
| --- | --- | --- | --- | --- | --- | --- | --- | --- |
|  | **Est.** | **SE** | **t-value** | **p** | **Est.** | **SE** | **t** | **p** |
| **Int.** | -5.20 | 0.94 | -5.53 | 3.17e-08 | -4.57 | 0.94 | -4.86 | 1.18e-06 |
| **ot1** | 0.52 | 14.82 | 0.04 | 0.97 | 42.98 | 14.82 | 2.90 | 0.003 |
| **ot2** | 194.45 | 23.78 | 8.18 | 2.22e-16 | 152.66 | 23.78 | 6.42 | 1.37e-10 |
| **ot3** | 66.93 | 20.64 | 3.24 | 0.0011 | 26.30 | 20.64 | 1.27 | 0.20 |
| **ot4** | -116.85 | 22.23 | -5.26 | 1.47e-07 | -125.31 | 22.23 | -5.64 | 1.74e-08 |
| **ot5** | -103.88 | 16.84 | -6.17 | 6.87e-10 | -106.18 | 16.84 | -6.31 | 2.88e-10 |

S4D Table. Model’s parameter estimates for sentence structures in quiet in comparison to noise.

|  | **quiet vs. noise for SVO** | | | | **quiet vs. noise for OVS** | | | | **quiet vs. noise for ambOVS** | | | |
| --- | --- | --- | --- | --- | --- | --- | --- | --- | --- | --- | --- | --- |
|  | **Est.** | **SE** | **t** | **p** | **Est.** | **SE** | **t** | **p** | **Est.** | **SE** | **t** | **p** |
| **Int.** | 0.89 | 0.94 | 0.95 | 0.34 | 0.23 | 0.94 | 0.24 | 0.81 | 0.86 | 0.94 | 0.92 | 0.36 |
| **ot1** | -1.50 | 14.82 | -0.10 | 0.92 | -21.99 | 14.82 | -1.48 | 0.14 | 20.47 | 14.82 | 1.38 | 0.17 |
| **ot2** | -8.44 | 23.78 | -0.36 | 0.72 | 17.88 | 23.78 | 0.75 | 0.45 | -23.91 | 23.78 | -1.01 | 0.31 |
| **ot3** | 37.22 | 20.64 | 1.80 | 0.07 | 23.05 | 20.64 | 1.12 | 0.26 | -17.58 | 20.64 | -0.85 | 0.39 |
| **ot4** | 32.32 | 22.23 | 1.45 | 0.15 | -1.12 | 22.23 | -0.05 | 0.96 | -9.57 | 22.23 | -0.43 | 0.67 |
| **ot5** | -22.43 | 16.84 | -1.33 | 0.18 | 16.56 | 16.84 | 0.98 | 0.33 | 14.27 | 16.84 | 0.85 | 0.40 |

S4E Table. Model’s parameter estimates for the comparison between sentence structures in quiet vs. noise.

|  | **SVO vs. OVS in quiet relative to SVO vs. OVS in noise** | | | | **SVO vs. ambOVS in quiet relative to SVO vs. ambOVS in noise** | | | | **OVS vs. ambOVS in quiet relative to OVS vs. ambOVS in noise** | | | |
| --- | --- | --- | --- | --- | --- | --- | --- | --- | --- | --- | --- | --- |
|  | **Est.** | **SE** | **t** | **p** | **Est.** | **SE** | **t** | **p** | **Est.** | **SE** | **t** | **p** |
| **Int.** | -0.66 | 1.33 | -0.50 | 0.62 | -0.03 | 1.33 | -0.02 | 0.98 | 0.63 | 1.33 | 0.48 | 0.63 |
| **ot1** | -20.49 | 20.96 | -0.98 | 0.33 | 21.96 | 20.96 | 1.05 | 0.29 | 42.46 | 20.95 | 2.03 | 0.04 |
| **ot2** | 26.32 | 33.63 | 20.78 | 0.43 | -15.47 | 33.63 | -0.46 | 0.65 | -41.79 | 33.63 | -1.24 | 0.21 |
| **ot3** | -14.17 | 29.18 | -0.49 | 0.63 | -54.80 | 29.18 | -1.88 | 0.06 | -40.63 | 29.19 | -1.39 | 0.16 |
| **ot4** | -33.43 | 31.44 | -1.06 | 0.29 | -41.89 | 31.44 | -1.33 | 0.18 | -8.46 | 31.44 | -0.27 | 0.79 |
| **ot5** | 38.99 | 23.81 | 1.64 | 0.10 | 36.70 | 23.81 | 1.54 | 0.12 | -2.29 | 23.81 | -0.10 | 0.92 |
